# An Extremely Rare Case of Coexisting Neuroendocrine Carcinoma and Tumor of the Whole Pancreas with Hepatoid Differentiation

**DOI:** 10.70352/scrj.cr.24-0058

**Published:** 2025-07-01

**Authors:** Dongha Lee, Keiko Kamei, Masaya Nakano, Katsuya Ami, Chihoko Nobori, Yuta Yoshida, Takaaki Murase, Atsushi Takebe, Takuya Nakai, Takaaki Chikugo, Ippei Matsumoto

**Affiliations:** 1 Department of Surgery, Faculty of Medicine, Kindai University, Osaka-Sayama, Osaka, Japan; 2 Department of Pathology, Faculty of Medicine, Kindai University, Osaka-Sayama, Osaka, Japan

**Keywords:** hepatoid carcinoma of the pancreas, neuroendocrine tumor, neuroendocrine carcinoma, hepatoid differentiation

## Abstract

**INTRODUCTION:**

Hepatoid carcinoma (HC) is a rare type of malignant tumor that shares similar serological, morphological, and immunohistochemical features with hepatocellular carcinoma. Pancreatic HC exhibits aggressive biological behavior and is classified as pure type, combined type, and mixed type. Unlike the pure type, the combined and mixed types refer to HC with other histological components. While the most common component is the neuroendocrine tumor (NET), no case of coexistence of neuroendocrine carcinoma (NEC) and NET with hepatoid differentiation has been reported. We herein report an extremely rare case of coexisting NEC and NET of the whole pancreas with hepatoid differentiation.

**CASE PRESENTATION:**

A 15-year-old woman had epigastric pain and impaired glucose tolerance, without any medical or family history. Computed tomography (CT) revealed a 6.4-cm mass in the pancreatic head, an enhanced mass throughout the pancreas, and a 1.0-cm liver lesion. Positron emission tomography (PET)/CT and somatostatin receptor scintigraphy (SRS) suggested a NET in the pancreatic body and tail, and a NEC in the pancreatic head. Biopsies confirmed NEC in the pancreatic head and liver with possible hepatoid differentiation. The patient underwent combination chemotherapy with carboplatin and etoposide. Due to the partial response achieved with chemotherapy, which led to significant tumor shrinkage on CT and no uptake on PET/CT in the pancreatic head tumor, the patient proceeded with conversion surgery, including a total pancreatectomy with portal vein resection and partial hepatectomy. However, serum α-fetoprotein (AFP) levels rapidly rose, and multiple liver metastases of NEC were detected on CT at 5 months after surgery. Liver metastases worsened despite further chemotherapy. The patient died 10 months after surgery.

**CONCLUSIONS:**

We herein present an extremely rare case of coexisting NET and NEC of the whole pancreas with hepatoid differentiation. Due to a remarkable response to chemotherapy, conversion surgery was performed. However, early recurrence of liver metastases accompanied by a rapid increase in serum AFP levels occurred, and the prognosis was poor. Pancreatic HC should be considered when encountering a bulky tumor of the pancreas with elevated serum AFP levels, and further case series and analysis are needed to determine the appropriate treatment strategy.

## Abbreviations

AFP
: α-fetoprotein
CA19-9
: carbohydrate antigen 19-9
CEA
: carcinoembryonic antigen
CT
: computed tomography
EUS
: endoscopic ultrasonography
EUS-FNA
: EUS-guided fine-needle aspiration
HC
: hepatoid carcinoma
HepPar-1
: hepatocyte paraffin 1
NEC
: neuroendocrine carcinoma
NET
: neuroendocrine tumor
PET
: positron emission tomography
SRS
: somatostatin receptor scintigraphy

## INTRODUCTION

HC is a rare type of malignant tumor that shares similar serological, morphological, and immunohistochemical features with hepatocellular carcinoma. After Ishikura et al.^1)^ first reported an HC in a primary gastric tumor in 1985, HC has been described in various organs: ovary, esophagus, duodenum, colon, pancreas, rectum, gallbladder, endometrium, uterine cervix, fallopian tube, lung, kidney, and urinary bladder. Since the first case of primary pancreatic HC was reported by Yano et al.^2)^ in 1999, several reports of pancreatic HC have been described. Pancreatic HC exhibits aggressive biological behavior^3,4)^ and is classified as pure type or combined type. Unlike the pure type, the combined type refers to HC with other histological components, such as NET, endocrine tumor, islet cell glucagonoma, or pancreatic ductal adenocarcinoma,^5,6)^ While the most common component is the NET, no case of coexistence of NEC and NET of the whole pancreas with hepatoid differentiation has been reported.

We herein report an extremely rare case of coexisting NEC and NET of the whole pancreas with hepatoid differentiation in a 15-year-old woman.

## CASE PRESENTATION

**Fig. 1:**
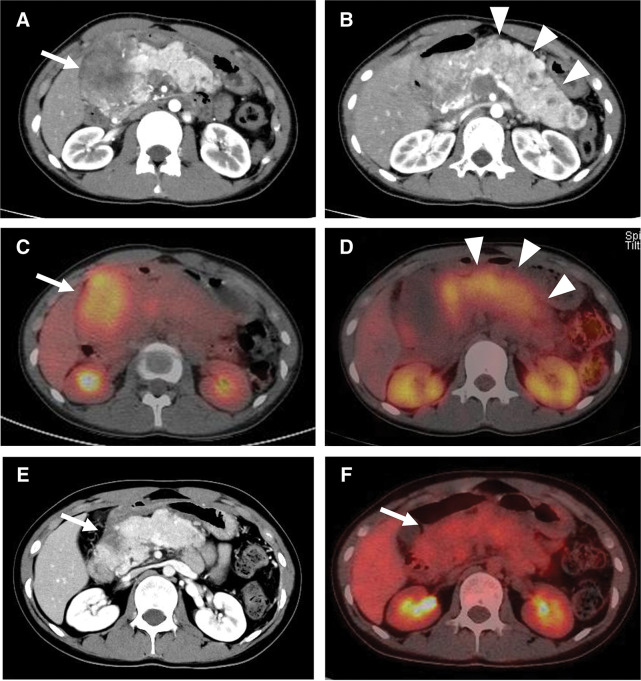
(**A**) CT shows a hypo-enhanced tumor in the pancreatic head (arrow). (**B**) CT demonstrates an enhanced tumor in the body and tail in the pancreas (arrowheads). (**C**) PET/CT shows intense accumulation in the pancreas head tumor (arrow), but not in the body and tail of the pancreas. (**D**) Somatostatin receptor scintigraphy shows negative accumulation in the pancreatic head mass, but significant accumulation in the body and tail of the pancreas (arrowheads). (**E**) CT image showing remarkable tumor shrinkage in the pancreatic head after chemotherapy (arrow). (**F**) PET/CT shows no accumulation in the pancreatic head tumor after chemotherapy (arrow).

**Fig. 2:**
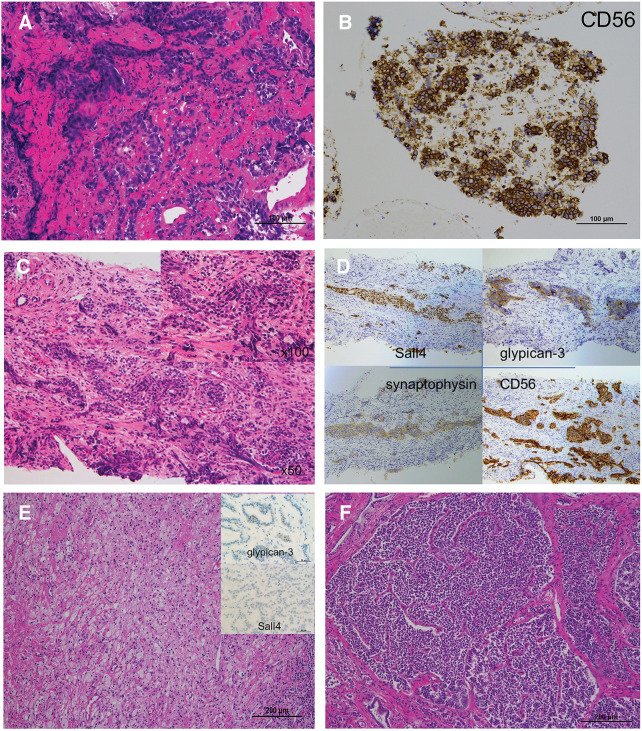
Microscopic findings of the EUS-FNA from the pancreatic head tumor. (**A**) HE staining shows a proliferation of small round cells with high nuclear-to-cytoplasmic ratios. (**B**) Immunohistochemical examination shows CD56 immunopositivity. Microscopic findings of the liver tumor biopsy. (**C**) Tumor cells, characterized by round to oval atypical nuclei and clear cytoplasm, are arranged in nests and proliferate within a fibrous stroma. (**D**) Immunohistochemical examinations shows immunopositivity for CD56, synaptophysin, Sall4, and glypican-3. Microscopic findings of the resected specimen. (**E**) Fibrosis is present in the pancreatic head, but there is no small-cell NEC. Immunohistochemistry of the resected pancreatic tumor shows weak positivity for glypican-3 and negativity for Sall4. (**F**) Microscopic findings of the resected specimen reveals a glandular or trabecular pattern (NET G2) throughout the entire pancreas (mostly the body and tail). EUS-FNA, endoscopic ultrasonography-guided fine-needle aspiration; HE, hematoxylin–eosin; NEC, neuroendocrine carcinoma; NET, neuroendocrine tumor

**Fig. 3:**
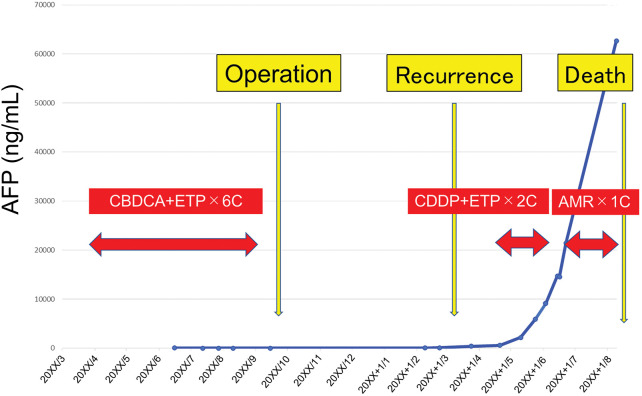
Changes in serum AFP levels during the treatment course. AFP, α-fetoprotein; AMR, amrubicin hydrochloride; CBDCA, carboplatin; CDDP, cisplatin; ETP, etoposide

A 15-year-old woman having epigastric pain and no specific medical or family history was referred to our hospital. Blood examination revealed no obvious abnormal findings; however, biochemical analysis showed impaired glucose tolerance (hemoglobin A1c, 7.6%). Contrast-enhanced CT revealed a hypo-enhanced mass measuring 6.4 cm in the pancreatic head, along with an enhanced mass throughout the entire pancreas, primarily affecting the body and tail (**Fig. 1A** and **1B**). Simultaneously, a hypo-enhanced mass measuring 1.0 cm, suspected to be metastatic, was found in the liver (segment 5). Gadolinium-ethoxybenzyl-diethylenetriamine pentaacetic acid (Gd-EOB-DTPA) MRI revealed no intrahepatic lesions other than the solitary lesion in segment 5. PET/CT showed intense accumulation in the pancreatic head mass, but not in the body and tail (**Fig. 1C**). SRS showed negative accumulation in the pancreatic head mass, but significant accumulation in the body and tail (**Fig. 1D**). Regarding the suspected liver metastatic lesion, PET/CT and SRS showed negative accumulation. Serum tumor markers demonstrated normal levels of CEA (1.3 ng/mL) and CA19-9 (30 U/mL). The pancreatic body and tail lesion was suspected to be NET based on the findings of contrast-enhanced CT and SRS. EUS was performed to identify characteristics and obtain histological findings. On EUS, the pancreatic head lesion was detected as a low echoic mass, and EUS-FNA was performed. EUS-FNA of the pancreatic head mass confirmed the presence of a small-cell NEC (**Fig. 2A**). Immunohistochemical analysis revealed that the neoplastic cells were positive for CD56, synaptophysin, and Rb, but negative for BCL-10, trypsin, and p53 (**Fig. 2B**). A liver tumor biopsy was performed at another hospital for a 2nd opinion after 1 course of chemotherapy for NEC. The liver biopsy for the mass revealed liver metastasis of NEC and potential HC differentiation (**Fig. 2C**). Immunohistochemical analysis revealed that the neoplastic cells were positive for CD56, synaptophysin, Sall4, and glypican-3, but negative for HepPar-1 and AFP (**Fig. 2D**). The Ki-67 values were 25% and 70% for the pancreatic head and liver masses, respectively. The pancreatic head lesion was diagnosed as NEC, the pancreatic body and tail lesion as NET, and the liver lesion as metastasis of NEC with possible HC differentiation. The patient underwent 5 cycles of combination chemotherapy with carboplatin and etoposide. After chemotherapy, a follow-up Gd-EOB-DTPA MRI showed a reduction in the size of the metastatic lesion, with no evidence of metastases in other regions. Due to the partial response achieved with chemotherapy, which led to significant tumor shrinkage on CT and no uptake on PET/CT in the pancreatic head tumor (**Fig. 1E** and **1F**), the patient proceeded with conversion surgery. This involved a total pancreatectomy with portal vein resection and partial hepatectomy. Pathological examination revealed the presence of NET G2 throughout the entire pancreas, with fibrosis in the pancreatic head (**Fig. 2E** and **2F**). In the NET component of the pancreatic body and tail, the mitotic count slightly exceeded the threshold of 2 per 10 high-power fields (HPFs), and the Ki-67 index was below 1%, supporting a classification of NET G2 according to the World Health Organization grading system. No small-cell NEC was found in the pancreas or liver specimens. Immunohistochemical staining of the resected pancreatic tumor tissue showed only weak positivity for glypican-3 and negativity for Sall4. However, metastatic lesions were observed in the regional lymph nodes, and histologically, they were composed of NEC components. Due to the lack of evidence supporting adjuvant chemotherapy, postoperative adjuvant chemotherapy was not initiated. The measurement of serum AFP levels was initiated during the administration of chemotherapy before surgery due to the possibility of HC. Serum AFP levels remained within the normal range from the start of measurements until recurrence; however, they rapidly increased to 554 ng/mL, and CT revealed multiple liver metastases of NEC at 5 months after surgery. Combination chemotherapy with cisplatin and etoposide was initiated, but was ineffective. The regimen was changed to amrubicin hydrochloride. However, liver metastases worsened, and the levels of serum AFP rose to 60000 ng/mL (**Fig. 3**). The patient died 10 months after surgery.

## DISCUSSION

Pancreatic HC is rare and associated with aggressive biological behavior.^3,4)^ Pancreatic HC can be categorized as pure type or combined type. Unlike pure type, combined type refers to HC with other histological components such as NET, endocrine tumor, islet cell glucagonoma, or pancreatic ductal adenocarcinoma.^5,6)^ In this study, pancreatic HC not only had a NET component but also a NEC component. There are few reports about pancreatic NET coexisting with other pancreatic tumors such as serous microcystic adenoma and pancreatic ductal adenocarcinoma.^7–9)^ However, to the best of my knowledge, there are no reports of coexistence of NET and NEC occupying the entire pancreas. This is the first report of coexistence of NET and NEC components with hepatoid differentiation occupying the entire pancreas.

The main clinical features of the 19 cases of pancreatic NEC with hepatoid differentiation, including our case, are summarized in **Table 1**.^10–13)^ The ages of patients range from 15 years to 90 years, with a median age of 51 years. The size of tumors ranges from 1 to 11 cm, with a median size of 6.3 cm. Pancreatic NEC with hepatoid differentiation was common in middle-aged patients with bulky tumors, and more than 70% of reported cases were curatively resected, but survival was poor (median survival time was 12 months).^12)^ In this study, the patient was the youngest case. Regarding subsequent treatment strategy after a remarkable response to chemotherapy, the option of continuing chemotherapy was considered. However, it was recognized that NEC frequently develops early resistance to chemotherapy in the cancer board meeting involving medical oncology, gastroenterology, and surgery. Furthermore, given the patient’s young age and her desire to pursue a curative approach, conversion surgery was planned at this time. As a result, no small-cell NEC was found in the pancreas or liver permanent specimen due to the response to chemotherapy. However, the patient’s serum AFP levels rapidly increased, and CT scans revealed multiple liver metastases of NEC 5 months after surgery. Despite subsequent chemotherapy, the patient died 10 months after surgery. Regarding the possibility of NET recurrence, although the ineffectiveness of chemotherapy does not entirely rule out this possibility, considering the differences in malignancy between NET and NEC, as well as the rapid progression observed during recurrence, we clinically consider this to be a recurrence of NEC with secondary resistance to chemotherapy. Even in cases where chemotherapy is effective, surgical indications should be carefully considered for pancreatic NEC with hepatoid differentiation.

**Table 1:** Main clinical features of 19 cases of pancreatic neuroendocrine carcinoma with hepatoid differentiation

Factors	No. of patients (*n* = 19)
Age (year), median (range)	51 (15–90)
Sex (male/female)	10/9
Tumor location, Ph/Pbt	7/12
Tumor size (cm), median (range)	6.3 (1–11)
Elevated serum AFP level, n (%)	9 (47%)
Concurrent liver metastases, n (%)	7 (36%)
Curative resection, n (%)	14 (74%)

AFP, α-fetoprotein; Ph, pancreatic head; Pbt, pancreatic body and tail

There are no standard criteria to diagnose pancreatic HC, but similarity to hepatocellular carcinoma is required, which could be supported by morphological and immunohistochemical findings.^2,3,12)^ Histologically, HC consists of medium to large cords of polygonal cells with abundant eosinophilic or clear cytoplasm and centrally located, vesicular nuclei in the sheet-like or trabecular portions. In this case, EUS-FNA of the pancreatic head mass led to a diagnosis of a small-cell NEC. After 1 course of chemotherapy with carboplatin (CBDCA) and etoposide (ETP), the patient sought a 2nd opinion at another institution. A liver biopsy performed at that institution resulted in a diagnosis suggestive of possible HC. The liver tumor cells, characterized by round to oval atypical nuclei and clear cytoplasm, were arranged in nests and proliferated within a fibrous stroma. The liver tumor exhibited features consistent with HC, which is known to frequently express neuroendocrine characteristics.^4,10,13)^ In contrast, the pancreatic tumor biopsy displayed a slightly different phenotype, with a stronger predominance of neuroendocrine features. These differences could be attributed to chemotherapy-induced modifications or intratumoral heterogeneity. Considering both morphological and pathological findings, the tumors were regarded as part of a single neoplastic process. Immunohistochemical studies play an important role in making a definitive diagnosis.^2,3,12,14)^ Pancreatic HC is often positive for hepatocellular immunohistochemical markers such as HepPar-1, glypican-3, and arginase-1. In this case, biopsy of the liver metastatic lesion indicated the possibility of HC, but the NEC component itself disappeared in the permanent specimen due to response to chemotherapy. Positive immunohistochemical staining helps in the diagnosis of HC, but the positivity rate is not high, ranging from 46% to 64%.^11,13)^ Although immunohistochemical staining was negative for HepPar-1 and AFP in this case, pancreatic HC was strongly suspected because of glypican-3 positivity in the liver tumor biopsy and a sudden elevation in serum AFP levels after recurrence. Immunohistochemical staining of the resected pancreatic tumor showed weak positivity for glypican-3 and negativity for Sall4, which may have been influenced by the favorable response to chemotherapy. However, the absence of definitive histological features in the resected specimen and the lack of pre-treatment AFP measurement or tissue biopsy at recurrence limit the diagnostic certainty. Therefore, the diagnosis of HC in this case should be interpreted as a clinicopathological hypothesis, supported by biopsy findings and clinical course, but not histologically confirmed.

## CONCLUSIONS

In summary, our report is the youngest reported case and the first to show hepatoid differentiation with coexisting NEC and NET throughout the entire pancreas. Due to the young age and response to chemotherapy, the patient underwent conversion surgery. Although no small-cell NEC was found in the pancreatic head and liver specimens due to the response to chemotherapy, the prognosis was poor and comparable to previous reports. Pancreatic HC should be considered when encountering a bulky tumor of the pancreas with elevated serum AFP levels, and further case series and analysis are needed to determine the appropriate treatment strategy.

## ACKNOWLEDGMENTS

We would like to thank Dr. Nobuyoshi Hiraoka of the Department of Pathology at the National Cancer Center Research Institute for reviewing the liver pathological specimens, and Dr. Hisato Kawakami of the Department of Medical Oncology at Kindai University for reviewing the clinical course.

## DECLARATIONS

### Funding

No funding was received in support of this work.

### Authors’ contributions

DL and IM conceived and designed this case report.

The remaining authors (KK, MN, KA, CN, YY, TM, AT, and TN) contributed to data collection and analysis.

TC re-evaluated and critically reviewed the pathological findings.

DL wrote the draft of the manuscript, and IM performed the critical revision to be published.

All authors took overall responsibility and guaranteed the scientific integrity of the manuscript.

All authors read and approved the final manuscript.

### Availability of data and materials

Not applicable.

### Ethics approval and consent to participate

This work does not require ethical considerations or approval. Informed consent to participate in this study was obtained from the patient.

### Consent for publication

Written informed consent was obtained from the patient and her family for the publication of the study and accompanying images.

### Competing interests

We have no conflict of interest to declare.
